# Deep learning techniques for cancer classification using microarray gene expression data

**DOI:** 10.3389/fphys.2022.952709

**Published:** 2022-09-30

**Authors:** Surbhi Gupta, Manoj K. Gupta, Mohammad Shabaz, Ashutosh Sharma

**Affiliations:** ^1^ Department of Computer Science and Engineering Department, SMVDU, Jammu, India; ^2^ Model Institute of Engineering and Technology, Jammu, India; ^3^ School of Computer Science, University of Petroleum and Energy Studies, Dehradun, India

**Keywords:** artificial intelligence, cancer, deep learning, gene expression, Rna-sequences

## Abstract

Cancer is one of the top causes of death globally. Recently, microarray gene expression data has been used to aid in cancer’s effective and early detection. The use of DNA microarray technology to uncover information from the expression levels of thousands of genes has enormous promise. The DNA microarray technique can determine the levels of thousands of genes simultaneously in a single experiment. The analysis of gene expression is critical in many disciplines of biological study to obtain the necessary information. This study analyses all the research studies focused on optimizing gene selection for cancer detection using artificial intelligence. One of the most challenging issues is figuring out how to extract meaningful information from massive databases. Deep Learning architectures have performed efficiently in numerous sectors and are used to diagnose many other chronic diseases and to assist physicians in making medical decisions. In this study, we have evaluated the results of different optimizers on a RNA sequence dataset. The Deep learning algorithm proposed in the study classifies five different forms of cancer, including kidney renal clear cell carcinoma (KIRC), Breast Invasive Carcinoma (BRCA), lung adenocarcinoma (LUAD), Prostate Adenocarcinoma (PRAD) and Colon Adenocarcinoma (COAD). The performance of different optimizers like Stochastic gradient descent (SGD), Root Mean Squared Propagation (RMSProp), Adaptive Gradient Optimizer (AdaGrad), and Adaptive Momentum (AdaM). The experimental results gathered on the dataset affirm that AdaGrad and Adam. Also, the performance analysis has been done using different learning rates and decay rates. This study discusses current advancements in deep learning-based gene expression data analysis using optimized feature selection methods.

## 1 Introduction

**FIGURE 1:**
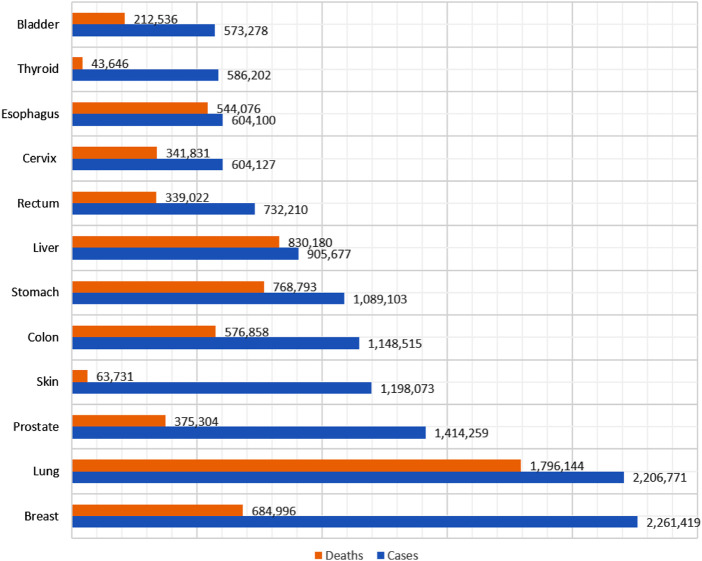
Cancer cases and deaths in 2020.

Cancer is one of the deadliest diseases, and with its increasing prevalence, early identification and treatment are critical (Sung et al., 2021) (Schiff et al, 2007; Reid et al, 2011). Lung cancer cases have been surpassed by female breast cancer cases and are one of the most often detected forms of cancer. Figure 1 shows the cancer cases and deaths in 2020.

About two-third of cases are detected at initial stages (Fotouhi et al, 2019; Id et al., 2021, Kashyap et al, 2022). The classification and identification of gene expression using DNA microarray data is an effective tool for cancer diagnosis and prognosis for specific cancer subtypes. AI-based learning algorithms are vital tools and the most often used way to achieve significant features of gene expression data and play an essential part in gene categorization. This article will give a review of some of those strategies from the literature and information on the various datasets on which these techniques are applied and their associated benefits and drawbacks. The most classic variants of deep learning, such as Convolution Neural Networks, Artificial Neural Networks, and Autoencoders, have been established as essential tools for clinical oncology research and can be used to drive decision-making regarding disease diagnosis and therapy. As time passes, sickness in general, and cancer in particular, grow increasingly complex and challenging to identify, analyze, and treat. Cancer research is a prominent topic of study in the medical world.

### 1.1 Distribution of articles

**FIGURE 2:**
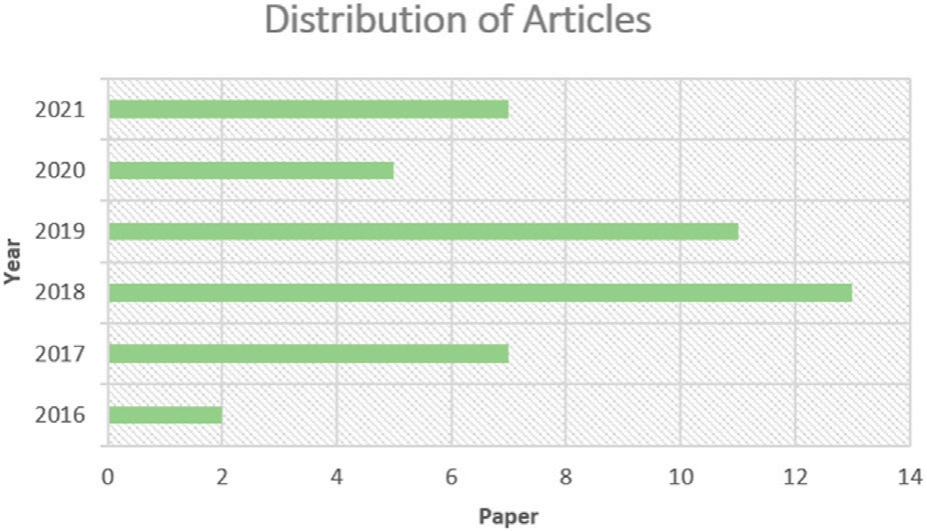
Year-wise Distribution of articles.

The selected articles for analysis have been published in last 5-years. Most of the research articles explored in this study have been published in 2018 and 2019. The articles that have explored gene expression data for cancer diagnosis/survival/stage prediction have been included in this study. Figure 2 presents the year-wise distribution of articles.

### 1.2 Contributions of study

The study contributes in a number of ways. Following are the significant contributions made by the study:

• This article reviews recent developments in deep learning-based feature selection techniques for gene expression data interpretation and offers an extensive review of Deep Learning architectures that have demonstrated success across a wide range of industries and are now used to help doctors identify various chronic conditions.
• In this work, we have compared the outcomes of several optimizers on a dataset of RNA sequences. The study’s deep learning system categorizes five types of cancer: colon cancer, lung adenocarcinoma, prostate cancer, invasive breast carcinoma, and kidney clear cell carcinoma (COAD).
• The efficiency of several optimizers, including adaptive gradient optimization (AdaGrad), stochastic gradient descent (SGD), root mean square propagation (RMSProp), as well as adaptive momentum (Adam). AdaGrad and Adam are more precise, according to the experimental findings discovered in the dataset. The performance of a variety of learning and decay rates was explored in the performance study.

### 1.3 Organization of paper

This paper is organized in a way that boosts the comprehensibility of the article. Second section gives the description of the significance of gene-expression analysis in cancer research. Section 2 gives description of search strategy used to select the articles for this study. Further Section 3 presents an overview of deep learning approaches where conventional approaches are discussed. Section 4 illustrates the importance of deep learning techniques in Cancer Prediction. Further, Section 5 embraces the literature of recent studies that have explored the deep learning strategies for gene section or survival prediction from microarray gene expression datasets. The article is discussed and concluded in Section 6 and Section 7, respectively. This study reviews and presents a comparative analysis of the previous studies. This article aims to analyze the concepts underlying deep learning-based classification algorithms used in healthcare.

## 2 Search strategy

**FIGURE 3:**
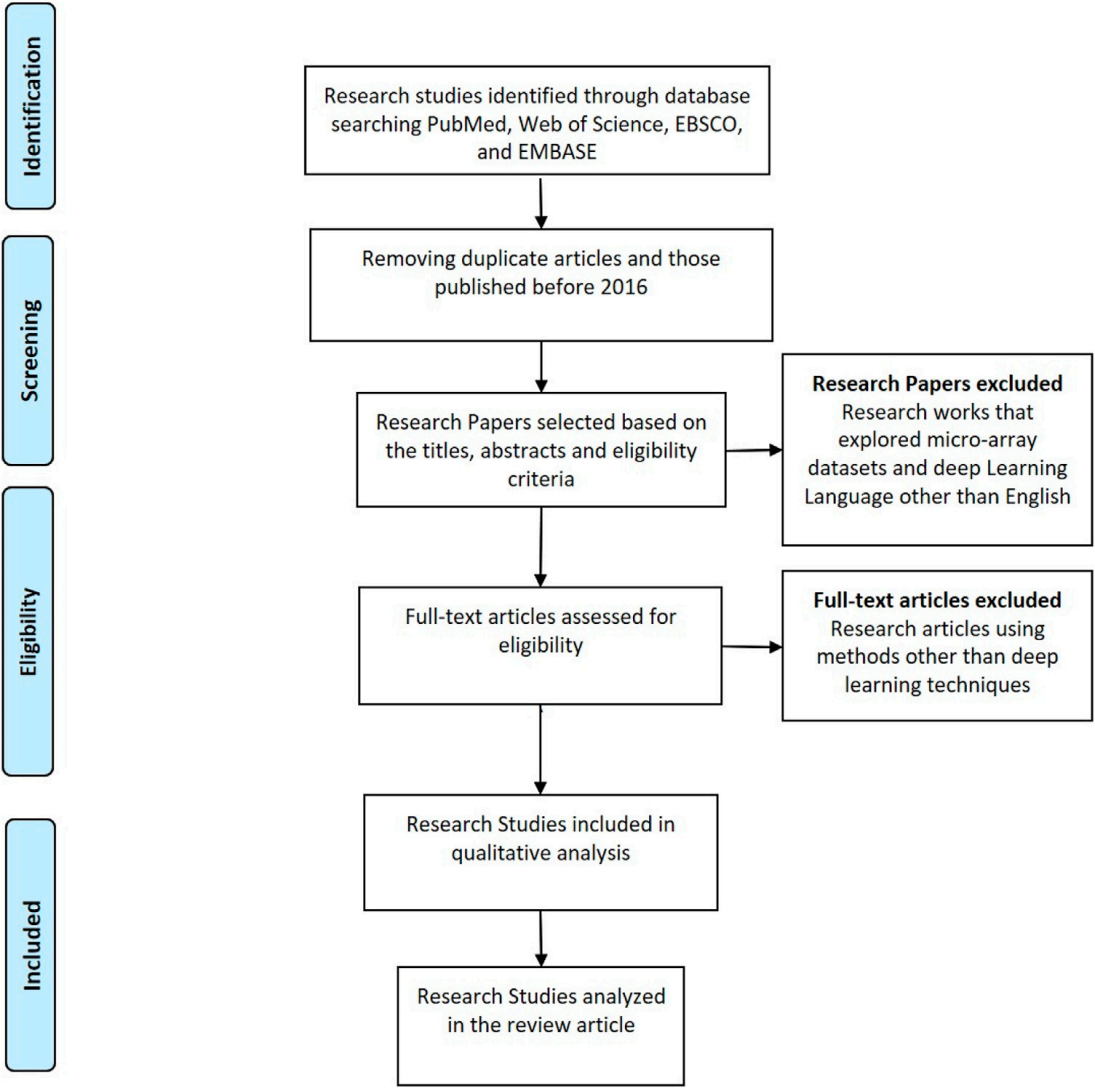
Prisma search strategy.

The search strategy used in this paper is Preferred Reporting Items for Systematic Reviews and Meta-Analyses (PRISMA) strategy. All the research studies selected for this systematic review have been extracted from databases like PubMed, Web of Science, EBSCO, and EMBASE. All the research articles that have been published before 2016 are excluded from the analysis. The keywords used for extraction of articles include “Deep Learning”, “Artificial Intelligence”, “Cancer”, “Micro-array analysis”, “gene-expression”, and combination of these keywords. The research articles that have focused on the optimization of gene selection using deep learning techniques have been included in the study. Figure 3 shows the PRISMA strategy flowchart.

## 3 Deep learning

**FIGURE 4:**
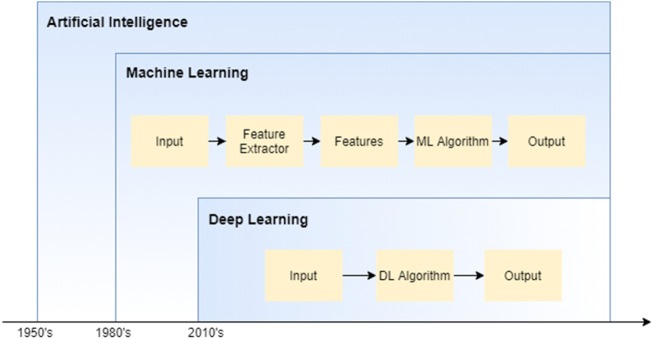
Artificial intelligence and sub-parts.

The Artificial intelligence is the idea of making innovative and intelligent machines. Machine learning is an artificial intelligence subset that aids in developing AI-driven applications. Deep learning is a subtype of machine learning that trains a model using large amounts of data and advanced methods. Figure 4 shows hierarchy of AI, Machine Learning, Deep Learning.

**FIGURE 5:**
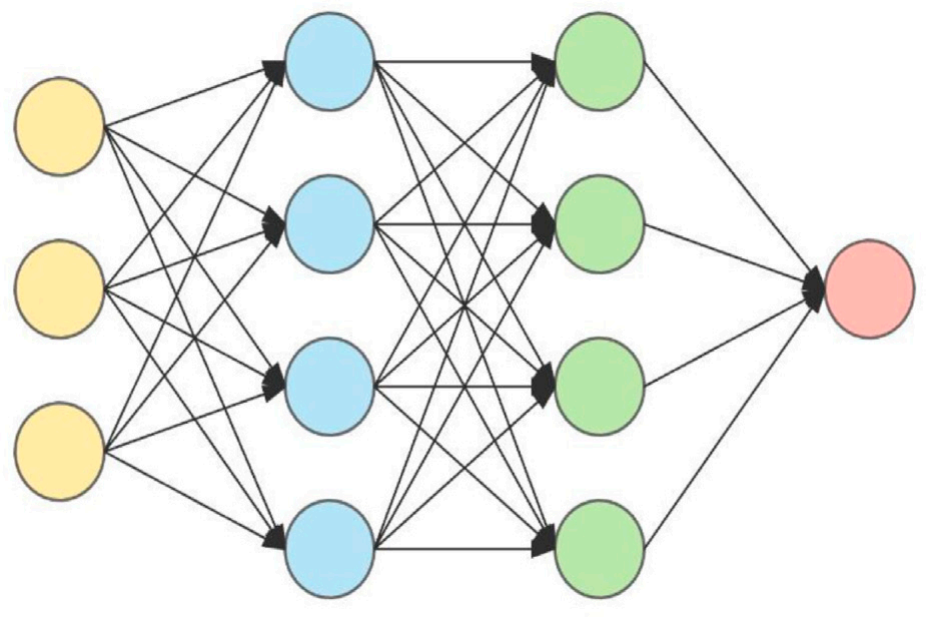
Artificial intelligence.

**FIGURE 6:**
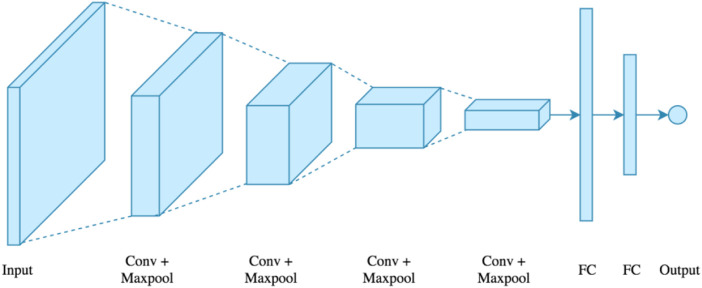
Convolutional neural network. • Long Short-Term Memory (LSTM) Network: Hochreiter and Schimdhuber collaborated to create the LSTM (Lecun et al, 2015), which is utilized in various applications. LSTMs were chosen by IBM primarily for voice recognition. The LSTM employs a memory unit known as a cell that may retain its value for an extended period and aids the device in remembering the most recent computed value. The memory unit, also known as a cell, comprises three gates that regulate the movement of data inside the unit. Figure 7 shows the logical structure of a LSTM model.

**FIGURE 7:**
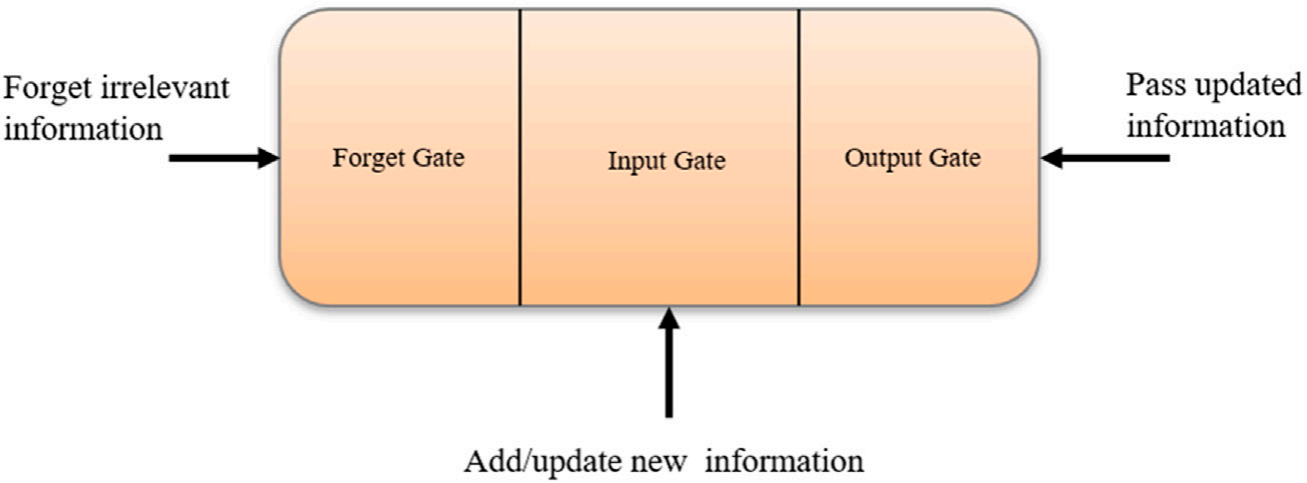
Long short-term memory.• The input port, also known as the gate, controls new data flow into the memory. • The forget gate forgets the irrelevant/unnecessary information. • The third port must regulate the information stored as output.

The significant differences between deep learning approaches and traditional learning are summarized in Table 1.

**TABLE 1:** Distinction between deep and traditional learning.

Feature	Traditional learning	Deep learning
Extraction and representation of features	Traditional learning relied on feature vectors that were manually created and were application-specific. In complexity, these characteristics are difficult to model	Deep learning approaches can learn characteristics from raw sensor data and determine the best pattern for enhancing recognition accurateness
Diversity and Generalization	Traditional learning relied on sensor data that had been tagged. Also, use dimensionality reduction strategies to focus on feature selection	Deep learning allows the extraction of intricate properties from complex data
Data preparations	Traditional learning derives features from sensors based on their appearance and active windows	Pre-processing and standardization of data are not required in deep learning
Changes in Activities’ Temporal and Spatial Dimensions	In traditional learning, handcrafted features are ineffective and unsuitable for resolving inter-class variability and inter-class linkages	Handcrafted characteristics with intra-class variability can be solved by using hierarchical features and translational invariant features
Model Training and Execution Time	In traditional training, small-sized data can also train the model and reduced computation time and space usage	Deep learning requires a vast amount of sensor datasets to avoid overfitting. It is accelerated using a graphics processing unit (GPU). It is also utilized to speed up computations

• Artificial Neural Networks: One of the most often used data modeling algorithms in medicine is neural networks. In the early 20th century, neural networks were developed (Daoud and Mayo, 2019). The primary goal of employing neural networks is to recognize patterns and conduct classification tasks. A human brain is used to represent the neural network system. The human brain is made up of millions of neurons that are all linked together. Figure 5 shows the representation of an artificial neural network.

Similarly, a neural network represents multiple neurons with a weight assigned to each link. These neurons act in parallel. During the learning stage, the network updates the weights for prediction of proper input to produce the output function (Gupta and Gupta, 2021b). Different optimization tasks are done by neural networks using different optimization techniques. Sigmoid optimization is mathematically given in Equation 1.

$$Sigmoid\left(a\right)=\frac{1}{1+e^{-a}}$$ (1)

The mathematical working of Hyperbolic Tangent (*Tanh*) optimization technique is given in Equation 2 *.*

$$\tanh\left(a\right)=\frac{2}{1+e^{-2a}}-1$$ (2)

The working of Rectilinear Unit (*Relu*) optimization technique is expressed in Eq. 3 *.*

relu(a)=max(0,a) (3)

Because of its adaptive character, altering the weights aids in the minimization of error. In contrast to basic modeling methods, neural networks have the advantage of predicting non-linear relationships. In the study of medical data, neural networks play a significant role such as medication development. The use of a neural network to predict cardiac disease is possible.

• Convolutional Neural Network (CNN): CNN is a multi-layer neural network based on the visual brain of animals. LeCun et al. constructed the first CNN. CNN’s major application areas include image processing and character recognition (Akkus et al, 2017; Zahras, 2018). In terms of construction, the initial layer recognizes features, however the intermediate layer recombines features to produce high-level input characteristics, followed by classification. The collected characteristics will then be pooled, which reduces their dimensionality. Convolution and pooling are the following steps, which are then put into a fully connected multi-layer perceptron. The last layer, known as the output layer, recognizes the image’s characteristics using back-propagation techniques (Gupta and Gupta, 2021a). Because of its unique properties, such as local connection and shared weights, CNN increases the system’s accuracy and performance. It outperforms all other deep learning techniques. In comparison to other types of architecture, it is the most often utilized. Figure 6 shows a convolutional neural network.

The cell’s weight can be utilized as a regulating factor. There is a requirement for a training approach known as Backpropagation through time (BPTT) that improves weight. For optimization, the technique requires network output error.

## 4 Deep learning in cancer prediction

Deep learning has been widely utilized to improve prognosis (Huang et al., 2020). Gene expression profiles, which describe the molecular state, offer enormous promise as a medical diagnostic tool. However, current training data sets have a minimal sample size for classification compared to the number of genes involved, and these training data constraints challenge specific classification techniques. One of the most important new clinical applications of microarray data is abnormality detection. Because of the high dimensionality, gene selection is a crucial step in enhancing the classification performance of expression data. As a result, better approaches for selecting functional genes for cancer prediction and detection are required. Microarray studies yield a massive quantity of gene-expression information from a single sample. The quantity of gene-expressions (features) to cases (samples) ratio is highly skewed, resulting in the well-known curse-of-dimensionality issue. In a single experiment, microarray technology generates hundreds of gene expressions. However, comparing the quantity of characteristics, the quantity of samples/patients is significantly lower (up to a few hundred) (several thousand). The limited number of samples (training data) provided is insufficient to create an efficient model from the given data. This is referred to as data scarcity.

Processing microarray gene expression data is a diverse field of computer science that includes graph analysis, machine learning, clustering, and classification. Microarray technology allows for the measurement of thousands of gene expressions in a single experiment. Gene expression levels aid in identifying linked genes and disease development, which aids in the early detection and prognosis of many forms of cancer.

## 5 Literature work

Using microarray gene expression patterns (Dwivedi, 2016), develop a framework of supervised machine learning approaches for discriminating acute lymphoblastic leukemia from acute myeloid leukemia. This classification was accomplished using an artificial neural network (ANN) (Tumuluru and Ravi, 2017). Using microarray gene expression patterns develop a framework of supervised machine learning approaches for discriminating acute lymphoblastic leukemia from acute myeloid leukemia. This classification was accomplished using an artificial neural network (ANN). In 2020, prostate cancer (Surbhi Gupta, 2021) was predicted using Multi-layer perceptrons and explored multiple data balancing techniques. Another recent study in 2021 (Gupta and Gupta, 2021b) predicted mesothelioma with 96% accuracy using ANN (Tumuluru and Ravi, 2017). presented an approach for cancer categorization based on gene-expression data. The logarithmic transformation pre-processed the gene expression data to reduce the classification’s complexity, while the Bhattacharya distance identified the most informative genes. The weight update in Deep Belief Neural Networks has estimated the average error using GOA and Gradient Descent.

The experimentation with colon and leukemia data demonstrates the proposed cancer classification’s efficacy. The accuracy rate of the proposed classification approach employing gene expression data is 0.9534, and 0.9666 detection rate.

Despite decades of research, clinical diagnosis of cancer and the identification of tumor-specific markers remain unknown (Danaee et al., 2017). offered a deep learning technique for cancer detection and identifying critical genes for breast cancer diagnosis using autoencoders. The error rates are computed using log loss function given in Equation 4.

Logloss=∑J(k)log(L(m))+(1−J(k))(log(1−L(m))) (4)

In the above equation, J(k) and L(m) represent prediction and target values (Cho et al., 2018). applied automated learning to search for survival-specific gene mutations in patients with lung adenocarcinoma (LUAD) using data from TCGA. Distinct feature selection methods were utilized to find survival-specific mutations in response to particular clinical variables. Kaplan-Meier survival analysis was performed on the extracted LUAD survival-specific mutations individually or in groups. Patient death was strongly associated with mutations in MMRN2 and GMPPA, whereas patient survival was associated with mutations in ZNF560 and SETX. In addition, DNAJC2 and MMRN2 mutations were associated with a substantial negative correlation with overall survival, but ZNF560 mutations were associated with a significant positive correlation with overall survival (Lin et al, 2018). tested the proposed SSAE model on three public RNA-seq data sets of three types of cancers.

A retrospective study (Lin et al., 2018) investigated the use of Deep Learning (DL) to predict acute myeloid leukemia (AML) prognosis. This study used 94 AML cases from the TCGA database. Age, ten common cytogenetic mutations, and the 23 most common mutations have been used as input data. Also, the results suggested feasible applications of deep learning (DL) in the prognostic prediction utilizing next-generation sequencing (NGS) data as proof-of-concept research.

Research work (Parvathavardhini and Manju, 2020) proposed a Neuro-Fuzzy approach for interpreting gene-expression data from microarray experiments. The analysis enabled the detection and classification of cancer, hence facilitating treatment selection and development. The proposed strategy was evaluated against three publicly available datasets of cancer gene expression. Also (Sevakula et al, 2018), proposed a cancer-verification transfer learning process in combination with autoencoders. The cross entropy function is used for optimizing the neural models. The cross entropy ( CE) is calculated using Equation 5.

$$CE=\frac{1}{k}\overset{k}{\underset{i=1}{\sum }}Y_{i}\log\left(X_{i}\right)+\left(1-Y_{i}\right)\left(\log\left(1-X_{i}\right)\right)$$ (5)

The term $X_{i}$ denotes the probability for i ^th^ instance and $Y_{i}$ represents all the truth values for k instances. The algorithm’s performance was evaluated on the GEMLeR repository dataset, and hence has significant implications for precision medicine.

(Xu et al., 2019b) employed numerous computational methods for classifying cancer subtypes have been presented. However, the majority of them create the model only using gene expression data. 2019 (Huynh et al, 2019). proposed a new support vector machine (SVM) classification model for gene expression based on features collected from a deep convolutional neural network (DCNN). The Equation 6 illustrates the working of CNN.

$$K\left[x,y\right]=\left(a*b\right)\left[x,y\right]=\underset{j}{\sum }\underset{k}{\sum }b\left[j,k\right]a\left[x-j,y-k\right]$$ (6)

Here a and b denote the input data and kernel respectively. Also, [x,y] denote the row and column indexes of resultant matrix

Nonetheless, it is characterized by highly high-dimensional data, which results in an over-fitting problem for the classifying model (Lin et al, 2018). purposed a novel way for incorporating deep learning into an ensemble approach that included numerous machine learning models. First, the study provided valuable gene data to five distinct categorization models using differential gene expression analysis. Then outputs of the five classifiers are then combined using a deep learning algorithm.

Significant bioinformatics research (Shon et al, 2021) has been undertaken in cancer research, and bioinformatics methodologies may aid in developing methods and models for early prediction of stomach cancer. This study aimed to build a CNN algorithm to analyze TCGA data. This study merged RNA-seq, and clinical data looked for and assessed potential genes employing the CNN model. In addition, this study performed learning and evaluated the status of cancer patients. The proposed model acquired an accuracy of 95.96 percent and a critical status accuracy of 50.51 percent. Despite overfitting due to the small sample size, reasonably accurate results for the sample type were achieved. This method can be used to forecast the diagnosis of stomach cancer, which comes in various forms and has a variety of underlying causes.

(Gupta and manoj, 2021) discovered that group algorithms for chronic disease diagnosis could be more effective than baseline algorithms. Additionally, it outlines many impediments to furthering the use of machine learning classification to detect illness. The proposed strategy achieved 98.5, 99, and 100% accuracy in this study. The disease datasets used in the study includes Diabetes, Cardiovascular Disease, and Breast Cancer. The algorithms used for the disease prediction are Group Algorithms, Stacked, and Neural Network.

(Abdollahi et al, 2021) proposed a novel strategy for reducing the number of features by utilizing an autoencoder. Each gene’s weight is determined as a consequence of our autoencoder model. The weights indicate the magnitude of each gene’s effect on survival probability. Our approach enhances survival analysis by speeding up the procedure, increasing prediction accuracy, and decreasing the calculated survival probability’s error rate. The error rates are computed using root mean squared error (*RMSE*). The mathematical formula of RMSE is given in Equation 7) where A and O represent actual and observed values respectively.

$$RMSE=\sqrt{\frac{\sum {\left(\left(A\right)-\left(O\right)\right)}^{2}}{N}}$$ (7)

### 5.1 Comparative analysis

Multiple studies aimed to investigate cancer prediction models. Table 2 presents the research analysis table.

**TABLE 2:** Research analysis.

Study	Cancer dataset	Objective	Technique	Acc
Dwivedi, (2016)	Leukemia	Cancer classification employing microarray gene-expression data using deep learning	ANN	98%
Yuan et al. (2016)	12 selected types of cancer	Cancer type classification using deep learning and somatic point mutations	DeepGene	94%
Motieghader et al., 2017	6 different cancer	Cancer classification using microarray data using genetic algorithm	Genetic Algorithm	94%
Aziz et al., 2017	5 gene microarray datasets	Microarray data classification using novel hybrid method	Artificial Bee Colony (ABC)	95%
Tumuluru and Ravi, (2017)	Colon and Leukemia data	Implementing deep neural networks for cancer classification	GOA-based DBN	95%
Extraction, (2017)	Breast cancer	Integrated deep neural networks to predict breast cancer	Deep-SVM	70%
Danaee et al. (2017)	Breast cancer	Relevant gene identification for better cancer classification	Stacked Denoising Autoencoder (SDAE)	98%
Urda and Moreno, (2017)	3 cancer databases	Investigating RNA-sequence gene expression data utilizing deep learning	Regularized linear model (standard LASSO) and two deep learning models	75%
Salman, (2018)	TCGA	Analyzing the Effect of meta heuristic iteration on the neural networks in cancer data	GA and FWA	98%
Ching et al. (2018)	TCGA RNA-Sequence data	Evaluating deep learning technique for tumor detection	Cox-nnet.	--
Cho et al. (2018)	TCGA LUAD	Examining the relationship between specific gene mutations and lung cancer survival	Information gain, chi-squared test	--
Xiao et al. (2018b)	RNA-sequence data sets of three cancers	Analyzing deep learning technique to predict cancer employing RNA sequence data	Sparse Auto-Encoder (SSAE)	98%
Lin et al. (2018)	TCGA Leukemia	Introduced deep learning to Predicting Prognosis of Leukemia	Stacked Autoencoders	83%
Alomari et al. (2018)	10 microarray datasets	Implementing a novel strategy for gene selection based on a hybrid technique	Hybrid Bat-inspired Algorithm	100%
Parvathavardhini and Manju, (2020)	Gene expression data of liver cancer	Cancer gene recognition using neuro-fuzzy approach	Neuro-Fuzzy method	96%
Ahn and Lee, (2018)	TCGA	Recognition of cancer tissues using RNA-Sequence data	Deep neural network (DNN)	99.7%
Kong and Yu, (2018)	Two RNA-seq expression datasets	Extracting features for RNA-Sequence data classification	Forest Deep Neural Network (fDNN)	90.4%
Guo et al., (2018)	Multiple Cancer datasets	Cancer subtype classification using RNA-Sequence gene expression data	BCD Forest	92.8%
Chen et al. , (2018)	mRNA datasets from the GDC repository	Cancer type recognition using neural network	Deep Learning models	98%
Sevakula et al. (2018)	36 datasets from the GEMLeR repository	Implemented transfer learning for molecular cancer classification	Sparse Autoencoders on gene expression data	98%
Joshi and Park, (2019)	LUAD	Lung Cancer Subtype Classification using deep learning model	Sparse Cross-modal Superlayered Neural Network	99%
Gao et al., (2019)	gene expression data	Cancer subtype prediction using gene expression data	Deep cancer subtype classification (DeepCC)	90%
Basavegowda and Dagnew, (2020)	8 microarray cancer datasets	Deep neural networks for classifying microarray cancer data	7-layer deep neural network architecture	90%
Huynh et al., (2019)	TCGA	Developed hybrid approach for classifying RNA-sequence data	Deep convolutional neural network (DCNN)	95%
Xu et al., (2019a)	RNA-seq gene expression data	Cancer subtype classification	Deep flexible neural forest (DFNForest)	76%
Xiao et al., (2018a)	LUAD, BRCA, and STAD	Cancer type prediction using deep learning model	ensemble-based approach	97%
Guia, (2019)	RNA-sequence data from Pan-Cancer Atlas	Cancer type Classification using RNA-sequence data	DeepGx Convolutional neural network (CNN)	95.65%
Huang et al. (2020)	TCGA cancers	Cancer survival prediction from RNA-sequence data	AECOX (AutoEncoder with Cox regression network)	--
Shon et al. (2021)	TCGA stomach cancer dataset	Stomach cancer prediction using gene expression data	CNN	96%
García-díaz et al., (2019)	5 types of cancer	Multiclass cancer classification of gene expression RNA-Sequence data	Extreme Learning Machine algorithm	98.81%
Kim et al., (2020)	TCGA	Cancer prediction using gene expression data	NN, SVM, KNN, RF	94%
Jerez et al., (2020)	31 Tumor types	Prediction of cancer survival using gene-expression data	Transfer learning with CNN	73%
Panda, (2017)	10 most common UCI Cancer datasets	Analyzed microarray cancer data using deep neural networks	Elephant search optimization based deep learning approach	92%
He and Luo, (2020)	15 different cancer types	Prediction of the tissue-of-origin of cancer types on basis of RNA-sequence data	a novel NN model	80%
Abdollahi et al., (2021)	Diabetes, heart, cancer dataset	Disease prediction model for the healthcare system	neural network-based ensemble learning	100%
Torkey et al., (2021)	RNA-seq data of three datasets	Cancer survival analysis for microarray dataset.	AutoCox and AutoRandom	98%
Wessels et al., (2021)	prostate cancer patients	Prediction of lymph node metastasis straight from tumor histology in prostate malignancy	convolutional neural network	62%
Chaunzwa et al., (2021)	311 NSCLC patients at Massachusetts General Hospital	Tumor detection using CT images	convolutional neural network	71%
Gupta, (2021)	cervical cancer dataset	Prediction of Cervical Cancer risk factors	Ensemble model	99.7%
Gupta and Gupta, (2021a)	five benchmark datasets	Cancer diagnosis analysis along with imbalanced classes	Stacked Ensemble Model	98%

## 6 Experimental results

This section holds the simulation results achieved using ANN model along with multiple optimizers like Stochastic gradient descent (SGD), Root Mean Squared Propagation (RMSProp), Adaptive Gradient Optimizer (AdaGrad), and Adaptive Momentum (AdaM). Also, the performance analysis has been done using different learning rates and decay rates.

### 6.1 Dataset analysis

TCGA dataset is available at https://archive.ics.uci.edu/ml/datasets/gene + expression + cancer + RNA-Seq. This dataset comprises data on five different forms of cancer, including kidney renal clear cell carcinoma (**KIRC**), Breast Invasive Carcinoma (**BRCA**), lung adenocarcinoma **(LUAD**), Prostate Adenocarcinoma (**PRAD**) and Colon Adenocarcinoma (**COAD**). The dataset consists of 20,531 attributes of 801 patients.

### 6.2 Optimization with multiple optimizers

**FIGURE 8:**
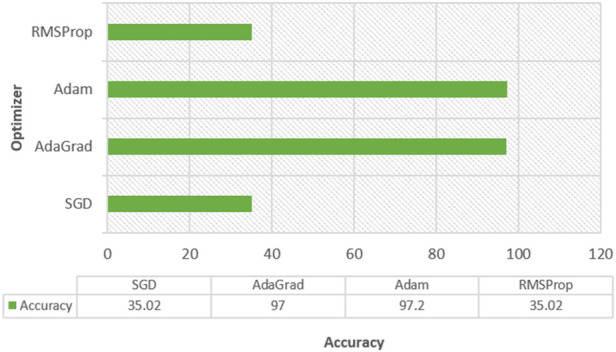
Accuracy of multiple optimizers.

The performance of multiple optimizers is analyzed and shown in Figure 8. From Figure 8, it is clear that both “Adam” and “Adagrad” performed the best on training and testing data.

The ANN model using SGD and rmsprop optimizer attained 35.3% on training data and 43.8% on test data. Both the Adam approaches performed well. Hence, we considered analyzing the performance of different parameters like learning rates and decay rates.

### 6.3 Optimization with learning rates

**FIGURE 9:**
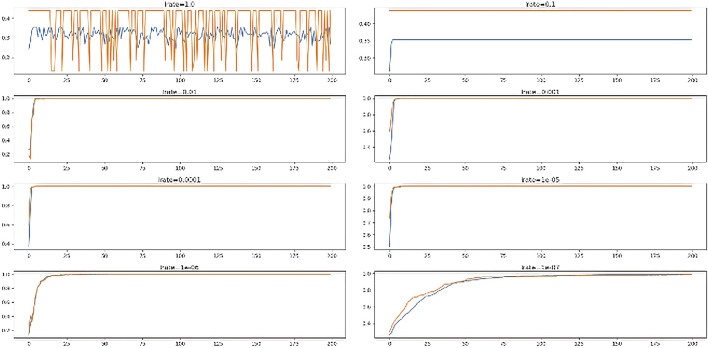
Performance of multiple learning rates.

The performance of ***ADAM*** optimizer using different learning rates is analyzed and shown in Figure 9.

From the figure, it is clear that learning rate (’0.01’, ‘0.001’, ‘0.0001’, ‘1e^−05^) performed the best on training and testing data. The ANN models performed worst (35% on train and 43.8% on test set) with slowest (lrate = “1.0”, “0.1”).

### 6.4 Optimization with decay rates

**FIGURE 10:**
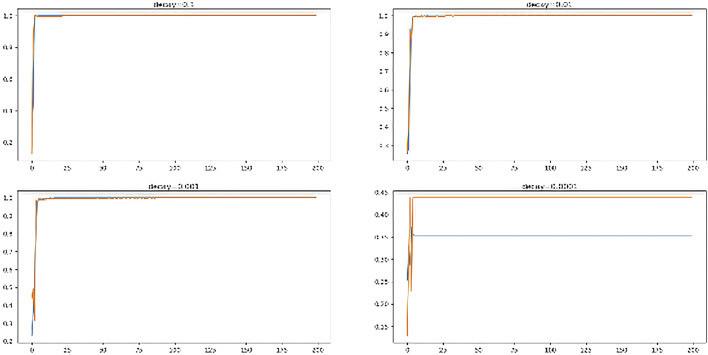
Performance of multiple decay rates.

The technique of learning rate decay (lrDecay) is used to train current neural networks. It begins with a high rate of learning and then decays several times. It has been demonstrated empirically to aid in both optimization and generalization. The performance of *ADAM* optimizer using different decay rates is investigated and revealed in Figure 10.

From the figure, it is clear that decay rate (“0.1”, “0.001”) performed the best on training and testing data. The ANN models performed worst (35.3% on train and 43.8% on test set) and (63.5% on train and 68.7% on test set) with decay rates “0.01” and “0.0001” respectively.

## 7 Discussion

**FIGURE 11:**
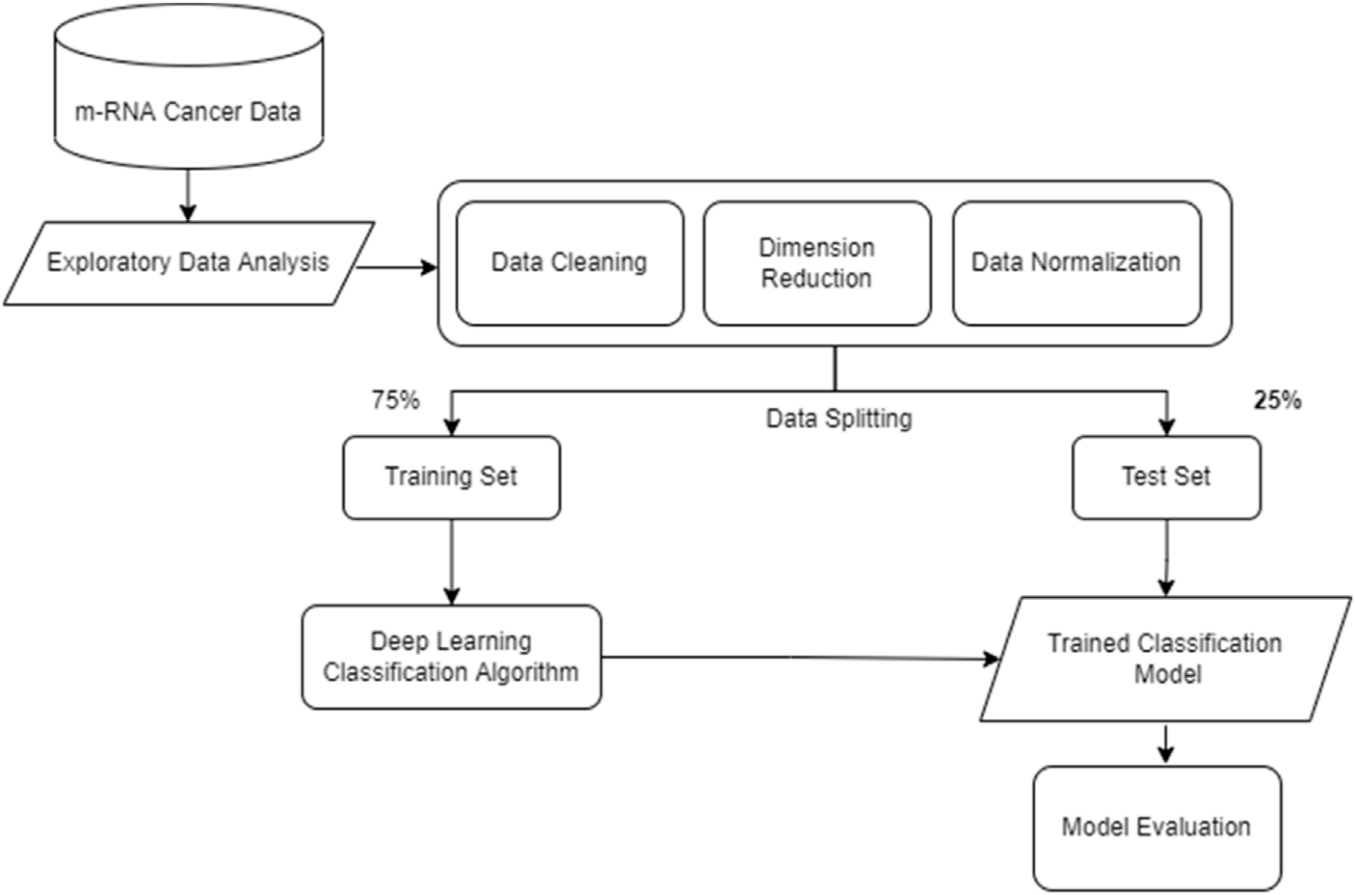
Deep learning for Cancer Classification.

Several strategies for gene selection in cancer categorization have been proposed in prior studies. The advent of deep learning has profoundly affected a wide variety of machine learning applications and research. Few of such studies (Gupta and Gupta, 2021a), (Gupta and Gupta, 2021b), (Gupta and Gupta, 2021c) are described in this section. The work flow used for classification of cancer data is shown in Figure 11.

Initially, the exploration of data is done and termed as “exploratory data analysis”. Further, data preprocessing steps are used like cleaning data, reducing dimension (feature reduction), normalizing the data. Further the next stage splits the preprocessed data into sets. The deep learning classification algorithm is trained on the training set for classification of data. The trained classification model is further evaluated on the test set. The evaluation of the data can express the accurateness of the model. The number of cancer cases is rapidly increasing. It is difficult to diagnose because the illness is frequently asymptomatic in its early stages. Early detection can increase the odds of a patient’s recovery and cure. Cancer is notoriously difficult to diagnose in its early stages and is prone to recurrence after treatment. Cancer classification is a crucial topic. One of the most effective methods for cancer classification is gene selection (Gupta and Gupta, 2021d). The task of choosing a set of genes that enhances classification accuracy is NP-Hard. Furthermore, making accurate and specific cancer diagnostic forecasts is quite tricky. Because of the nonspecific symptoms and imprecise scans, certain tumors are more challenging to diagnose in their early stages. As a result, improving the prediction model in diagnostic cancer research is vital. Furthermore, most cancer research articles have increased dramatically, particularly those that use deep learning methodologies (Shimizu and Nakayama, 2020). Again, the present research shows that traditional analysis techniques (Akkus et al., 2017; Ronoud and Asadi, 2019; Chaunzwa et al., 2021) aid in improving the prediction accurateness and is frequently applied in healthcare sector. Its success is since it enables the discovery of highly complicated non-linear correlations between characteristics; and the extraction of information from unlabeled data unrelated to the situation at hand. Statistical studies demonstrate that deep learning models outperform numerous widely used cancer categorization algorithms.

Several academics have investigated automated learning methodologies; however, these approaches still have several flaws that make cancer classification difficult. Specific machine learning algorithms have been found incapable of exploiting unstructured data in cancer classification. CNNs are particularly appropriate for analyzing a wide range of unstructured data. This capability enabled deep learning algorithms to take an active role in the early diagnosis of cancer through data classification. Deep learning approaches have achieved high accuracy and other statistical characteristics. Deep Learning has succeeded in various domains, including image, video, audio, and text processing. Deep Learning faces a unique problem in gene expression analysis for various cancer detection and prediction tasks to define appropriate biomarkers for different cancer subtypes. Despite several research studies on multimodal treatment approaches, survival times remain short. The gathering of significant genes that can increase accuracy can provide adequate guidance in early cancer detection. Cancer can be classified into several subgroups. However, it is a complex task because of the vast number of genes and the comparatively few experiments in gene expression data (Kumar et al, 2021). Cancer identification from microarray gene expression data presents a significant difficulty due to the small sample size, high dimensionality, and complexity of the data (Dargan et al, 2020). There is a need for rapid and computationally efficient methods to address such issues. This study briefly explores the research studies that employed deep learning architectures that selected the most relevant genes for cancer prediction using gene expression data. Although Deep Learning has had success in various domains, it has yet to be thoroughly explored in genomics, notably in genomic cancer.

## 8 Conclusion

Cancer has become one of the top causes of death worldwide in recent years. As a result, increasing research is being done to determine the most effective diagnosing and treating cancer. However, cancer treatment faces numerous obstacles, as possible causes of cancer include genetic problems or epigenetic modifications in the cells. RNA sequencing is a substantial approach for assessing gene expression in model organisms and can provide information for bio-molecular cancer diagnosis. Microarray gene expression profiles can be used to classify tumors efficiently and effectively. Predicting various tumors is a significant problem, and offering accurate predictions would be highly beneficial in delivering better therapy to patients. The advent of deep learning approaches is critical for improving patient monitoring, as it can aid clinicians in making decisions regarding deadly diseases. Furthermore, Gene expression data are utilized to develop a classification model that will help cancer treatment. Classification of cancer subtypes is critical for effective diagnosis and individualized cancer treatment. The article concludes that the recent advances in high-throughput sequencing technology have resulted in the quick generation of multi-omics data from the same cancer sample. Thus, deep learning-based molecular illness classification holds considerable promise in the realm of genomics, particularly concerning gene microarray data.

## Data Availability

The original contributions presented in the study are included in the article/supplementary material, further inquiries can be directed to the corresponding author.
